# Supplementary material for: CRISPR-Enabled Autonomous Transposable Element (CREATE) for RNA-based gene editing and delivery
Source: EMBO Rep. 2025 Jan 9;26(4):1062–83. doi: 10.1038/s44319-024-00364-7 (PMC11850887; doi:10.1038/s44319-024-00364-7)
Supplement: Supplementary file 1 — Appendix [file 44319_2024_364_MOESM1_ESM.pdf]

Appendix Table of Content

Appendix Figures

Appendix Figure S1. Allele plots of PBS1 and PBS2 junctions from NGS analysis of AAVS1 locus edited cells **(Page 2)**

Appendix Figure S2. Allele plots of PBS1 and PBS2 junctions from NGS analysis of HEK3, PRNP and IDS loci edited cells **(Page 3)**

Appendix Table

Appendix Table S1. Target hybridization sequencing results for AAVS1 edited samples. (**Page 4)**

Appendix Table S2. Sequences of PBS sites and payloads used in this study. (**Page 5)**

Appendix Table S3. Sequences of primers used for genomic DNA amplification. (**Page 6)**

Appendix Table S4. Sequences of primers used for PCR to detect off-target insertion. (**Page 7)**

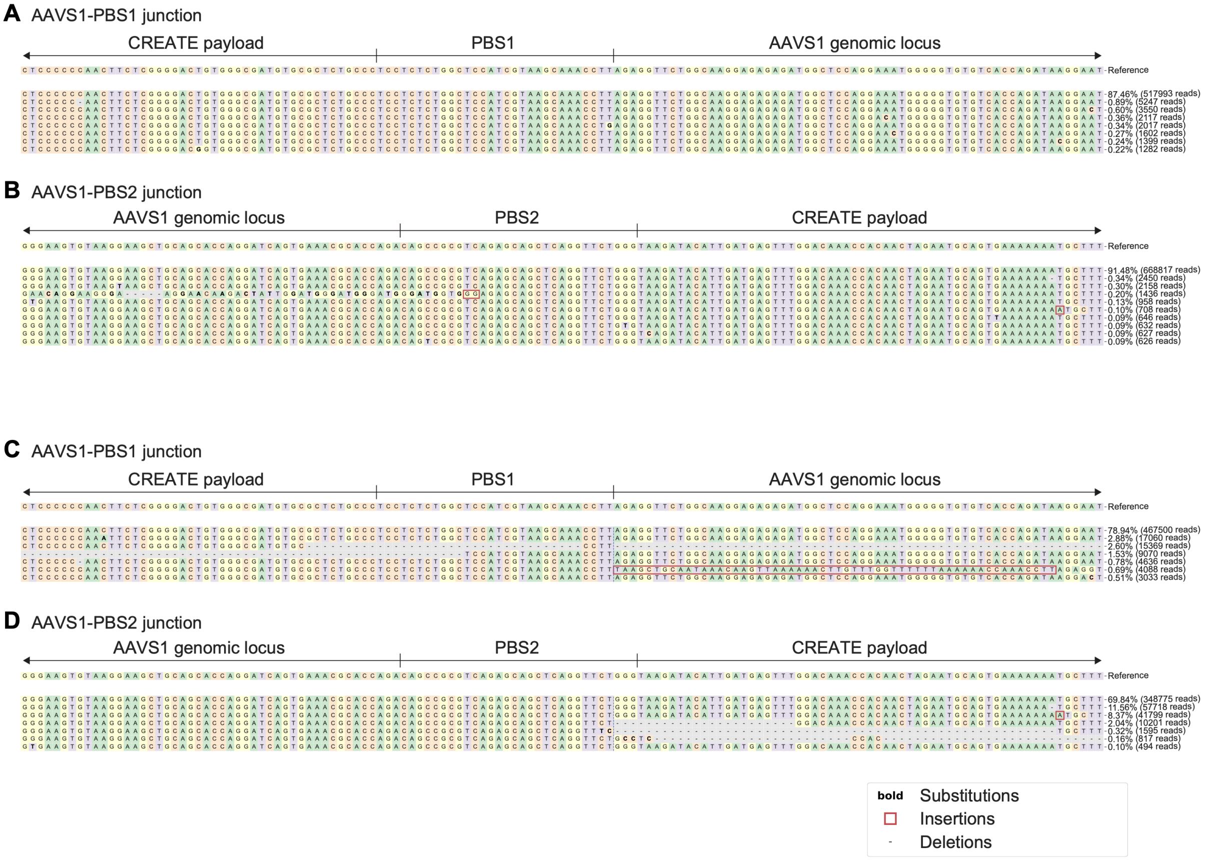

Appendix Figure S1. Allele plots of PBS1 and PBS2 junctions from NGS analysis of AAVS1 locus edited cells.

(**A**) and (**B**) are from AAVS1 (30 bp PBS) Exp1 edited cells. (**C**) and (**D**) are from AAVS1 (30 bp PBS) Exp2 edited cells.

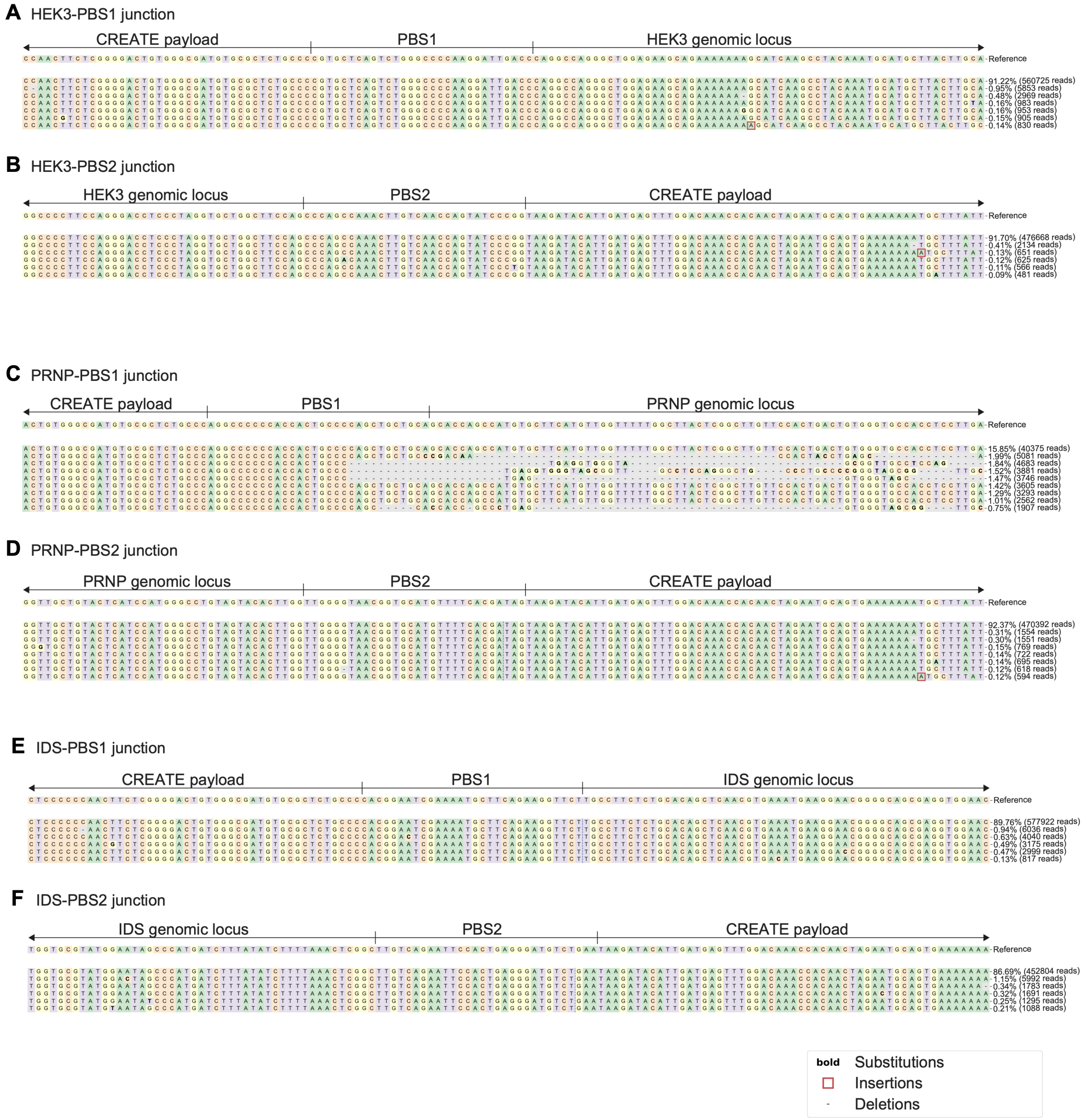

Appendix Figure S2. Allele plots of PBS1 and PBS2 junctions from NGS analysis of HEK3, PRNP and IDS loci edited cells.

Appendix Table S1. Target hybridization sequencing results for AAVS1 edited samples

**Chromosome**	**Location**	**Strand**	**Total_Reads**	**Annotation**
chr17	38905912	+	7	Endogenous Human EF1α sequence
chr19	55115486	-	26904	On-target insertion at AAVS1 (chr19: 5515280 – 5515880)
chr19	55115589	+	15692	
chr19	55115800	-	132	
chr19	55115148	-	8	
chr19	55115690	-	8	
chr19	55115285	-	5	
chr2	32916556	-	21	Mis-alignment to repetitive sequence
chr2	32916333	-	8	Mis-alignment to repetitive sequence
chr5	14653390	+	8	Endogenous Human EF1α pseudo gene 13 sequence
chr6	73521212	+	134	Endogenous Human EF1α sequence
chr6	73521001	-	117	

Identified potential insertion with >5 reads at a specific location are analyzed

Appendix Table S2. Sequences of PBS sites and payloads used in this study.

**EF1α-GFP payload in sense direction (1083 bp)**
EF1α core promoter-GFP-SV40 polyA signal<br>GGGCAGAGCGCACATCGCCCACAGTCCCCGAGAAGTTGGGGGGAGGGGTCGGCAATTGATCCGGTG<br>CCTAGAGAAGGTGGCGCGGGGTAAACTGGGAAAGTGATGTCGTGTACTGGCTCCGCCTTTTTCCCGA<br>GGGTGGGGGAGAACCGTATATAAGTGCAGTAGTCGCCGTGAACGTTCTTTTTCGCAACGGGTTTGCC<br>GCCAGAACACAGGGTTTAGTGAACCGTCAGATCCCGCCACCATGGTGAGCAAGGGCGAGGAGCTGT<br>TCACCGGGGTGGTGCCCATCCTGGTCGAGCTGGACGGCGACGTAAACGGCCACAAGTTCAGCGTGT<br>CCGGCGAGGGCGAGGGCGATGCCACCTACGGCAAGCTGACCCTGAAGTTCATCTGCACCACCGGCA<br>AGCTGCCCGTGCCCTGGCCCACCCTCGTGACCACCCTGACCTACGGCGTGCAGTGCTTCAGCCGCTA<br>CCCCGACCACATGAAGCAGCACGACTTCTTCAAGTCCGCCATGCCCGAAGGCTACGTCCAGGAGCGC<br>ACCATCTTCTTCAAGGACGACGGCAACTACAAGACCCGCGCCGAGGTGAAGTTCGAGGGCGACACC<br>CTGGTGAACCGCATCGAGCTGAAGGGCATCGACTTCAAGGAGGACGGCAACATCCTGGGGCACAAG<br>CTGGAGTACAACTACAACAGCCACAACGTCTATATCATGGCCGACAAGCAGAAGAACGGCATCAAGG<br>TGAACTTCAAGATCCGCCACAACATCGAGGACGGCAGCGTGCAGCTCGCCGACCACTACCAGCAGA<br>ACACCCCCATCGGCGACGGCCCCGTGCTGCTGCCCGACAACCACTACCTGAGCACCCAGTCCGCCCT<br>GAGCAAAGACCCCAACGAGAAGCGCGATCACATGGTCCTGCTGGAGTTCGTGACCGCCGCCGGGAT<br>CACTCTCGGCATGGACGAGCTGTACAAGTAAAACTTGTTTATTGCAGCTTATAATGGTTACAAATAAA<br>GCAATAGCATCACAAATTTCACAAATAAAGCATTTTTTTCACTGCATTCTAGTTGTGGTTTGTCCAAAC<br>TCATCAATGTATCTTA

**CREATE constructs**	**PBS1 sequence (5’-3’)**	**RC-PBS2 sequence (5’-3’)**
AAVS1_90-bp replacement_17-bp PBS	TCCTCTCTGGCTC	GCAGCTCAGGTTCTGGG
AAVS1_90-bp replacement_30-bp PBS	TCCTCTCTGGCTCCATCGTAAGCAAACCTT	CAGCCGCGTCAGAGCAGCTCAGGTTCTGGG
AAVS1_90-bp replacement_50-bp PBS	TCCTCTCTGGCTCCATCGTAAGCAAACCTTAGAGGTTCTGGCAAGGAGAG	GATCAGTGAAACGCACCAGACAGCCGCGTCAGAGCAGCTCAGGTTCTGGG
AAVS1_90-bp replacement_17-bp PBS reverse	CCCAGAACCTGAGCTGC	GAGCCAGAGAGGA
AAVS1_481-bp replacement_17-bp PBS	CAGGGGGCTGGAAGAGC	GCAGCTCAGGTTCTGGG
AAVS1_976-bp replacement_17-bp PBS	TCCTCTCTGGCTC	GCAGCTCAGGTTCTGGG
HEK3_90-bp replacement_30-bp PBS	CGTGCTCAGTCTGGGCCCCAAGGATTGACC	CCCAGCCAAACTTGTCAACCAGTATCCCGG
PRNP_72-bp replacement_30-bp PBS	AGGCCCCCCACCACTGCCCCAGCTGCTGCA	TTGGGGTAACGGTGCATGTTTTCACGATAG
IDS_70-bp replacement_30-bp PBS	CACGGAATCGAAAATGCTTCAGAAGGTTCT	TTGTCAGAATTCCACTGAGGGATGTCTGAA

Appendix Table S3. Sequences of primers used for genomic DNA amplification.

**Target**	**Forward primer (5'-3')**	**Reverse primer (5'-3')**
AAVS1-PBS1 junction	CGGAGCCAGTACACGACATC	TCCCAGGGCCGGTTAATGTG
AAVS1-PBS2 junction	GGGCTGGCTACTGGCCTTAT	ATCACTCTCGGCATGGACGA
PRNP-PBS1 junction	CGGAGCCAGTACACGACATC	CTGGAGGCAACCGCTACC
PRNP-PBS2 junction	GTGGTTGTGGTGACCGTGT	ATCACTCTCGGCATGGACGA
HEK3-PBS1 junction	CGGAGCCAGTACACGACATC	TGATGTGGGCTGCCTAGAAA
HEK3-PBS2 junction	GCCCTGAGATCTTTTCCTCTGT	CAACGAGAAGCGCGATCACA
IDS-PBS1 junction	AAAGAACGTTCACGGCGACT	CTGTTTCAGGCAGGCAATCC
IDS-PBS2 junction	GTTGGCAAAACTCAAGGCATCA	ACATGGTCCTGCTGGAGTTCG
AAVS1 full-length	GGGCTGGCTACTGGCCTTAT	TCCCAGGGCCGGTTAATGTG
HEK3 full-length	GCCCTGAGATCTTTTCCTCTGT	TGATGTGGGCTGCCTAGAAA
PRNP full-length	CAACCACGCGCTCCATCATC	CTGGAGGCAACCGCTACC
IDS full-length	GTTGGCAAAACTCAAGGCATCA	CTGTTTCAGGCAGGCAATCC

Note: junction primers are for Illumina short-read NGS; full-length primers are for Nanopore long-read NGS

Appendix Table S4. Sequences of primers used for PCR to detect off-target insertion.

Name	Target sequence	Location	GFP insertion forward primer (5'-3')	Genomic reverse primer (5'-3')	Genomic forward primer (5'-3')	Genomic amplicon size (bp)[#]	Predicted off-target amplicon (bp)[##]
AAVS1_nicking_PBS1	GATGGAGCCAGAGAGGATCC	chr19:-55115573	n/a	n/a	n/a	n/a	n/a
PBS1_off_target_1	GATGAAGTCAGAGAGGATCC	chr20:-2451191	GCTTGCCGTAGGTGGCAT	CACTCTGGCGTTAAAGGAGCA	GAAGTCACCCCCGAATCAGC	649	427
PBS1_off_target_2	CACGGAGCCAGGGAGGATCC	chr9:-127595186	GCTTGCCGTAGGTGGCAT	CAGGATCTTACCCTGCCATGA	AGACTCGGAGCTCAAACTGC	439	453
PBS1_off_target_3	GCTGGAGGCAAGAGAGGATCC	chr11:-75540340	GCTTGCCGTAGGTGGCAT	CATCACAATGCCCCAGGACT	GTCCCTTCACAGACCATGCG	757	513
PBS1_off_target_4	GATGAATGCTGAGAGGATCC	chr1:-93567284	GCTTGCCGTAGGTGGCAT	GCCGCTTGTGACGTTAATGG	ACCTCCTCATCTTGGCACATC	779	529
PBS1_off_target_5	GTGGGAGGCAGAGAGAATCC	chr1:-11579676	GCTTGCCGTAGGTGGCAT	GGCATTCCCTGTGAGAGTGT	GCCCTCTGTCAATAACCGCT	741	667
AAVS1_nicking_PBS2	GCAGCTCAGGTTCTGGGAGA	chr19:+55115469	n/a	n/a	n/a	n/a	n/a
PBS2_off_target_1	GAGGCTCCGGTTCTGGGAGA	chr1:+198494971	CGGGATCACTCTCGGCATGG	GCAGAGCTACCGGCTCCATG	CCTTTCACTGTGAACCTCATTGTTAGC	559	460
PBS2_off_target_2	TCAGTTCAGGTTCTGGAAGA	chr10:+6483525	GCATGGACGAGCTGTACAAGTAAAA	GCCCTCGGGGTCCCTATTTA	CCTTGATGTGGCATTATTAACAGTGC	662	556
PBS2_off_target_3	GCTGGCTCTGGTTCTGGGAGA	chr11:+132450011	GCATGGACGAGCTGTACAAGTAAAA	GCACCAACAGGTTCAGAGGT	GTGCTTTGGAGGACGACCAG	429	521
PBS2_off_target_4	GCCCCTCGGGTCCTGGGAGA	chr19:+44516904	GCATGGACGAGCTGTACAAGTAAAA	CGATTCCCCATATAGCTCACTCC	GGTGCATCACGTTTGGCTTT	953	809
PBS2_off_target_5	GCATTTCTGTTTCTGGGAGA	chr2:+30765077	CGGGATCACTCTCGGCATGG	GGCACGTGACCATCCCTTAAT	GGAACGCTTCCAATTCACCC	567	577

[#] Genomic amplicon size: If no off-target insertion happens, the PCR amplification using corresponding Genomic forward primer and Genomic reverse primer will produce PCR band of indicated size.
[##] Predicted off-target amplicon size: If off-target insertion happens based on the prediction, the PCR amplification using GFP insertion forward primer and Genomic reverse primer will produce PCR band of indicated size.
