# Supplementary material for: CRISPR-Enabled Autonomous Transposable Element (CREATE) for RNA-based gene editing and delivery
Source: EMBO Rep. 2025 Jan 9;26(4):1062–83. doi: 10.1038/s44319-024-00364-7 (PMC11850887; doi:10.1038/s44319-024-00364-7)
Supplement: Supplementary file 8 — Expanded View Figures [file 44319_2024_364_MOESM8_ESM.pdf]

Expanded View Figures

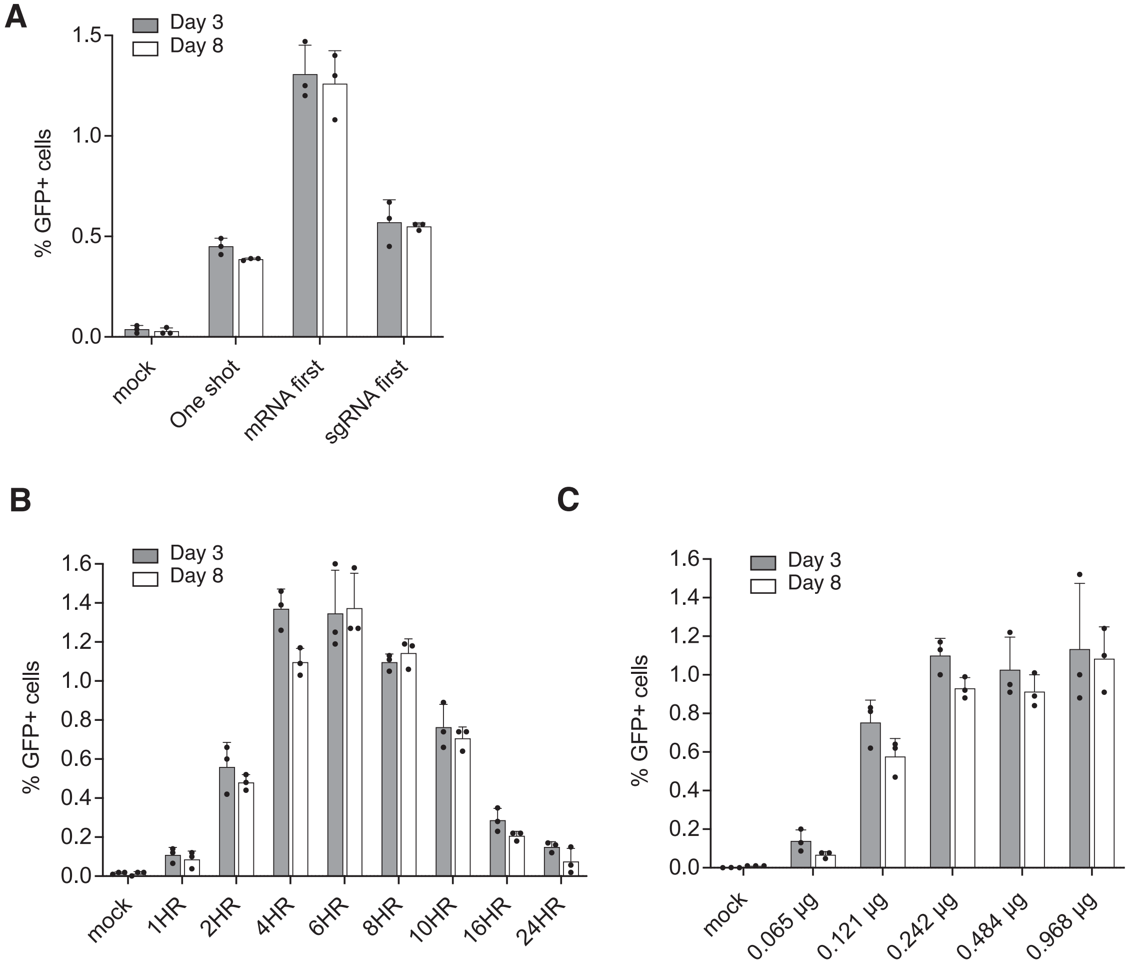

Figure EV1. Optimization of transfection protocol to improve CREATE editing efficiency.

(**A**) Optimization of transfection protocol improved editing efficiency. Protocol 1 (one shot), Protocol 2 (mRNA first) and Protocol 3 (sgRNA first). (**B**) Optimization of the incubation time between mRNA and sgRNA transfections. (**C**) Optimization of the total amount of sgRNAs transfected. Data are mean ± SD (*n* = 3 biological replicates).

© The Author(s)

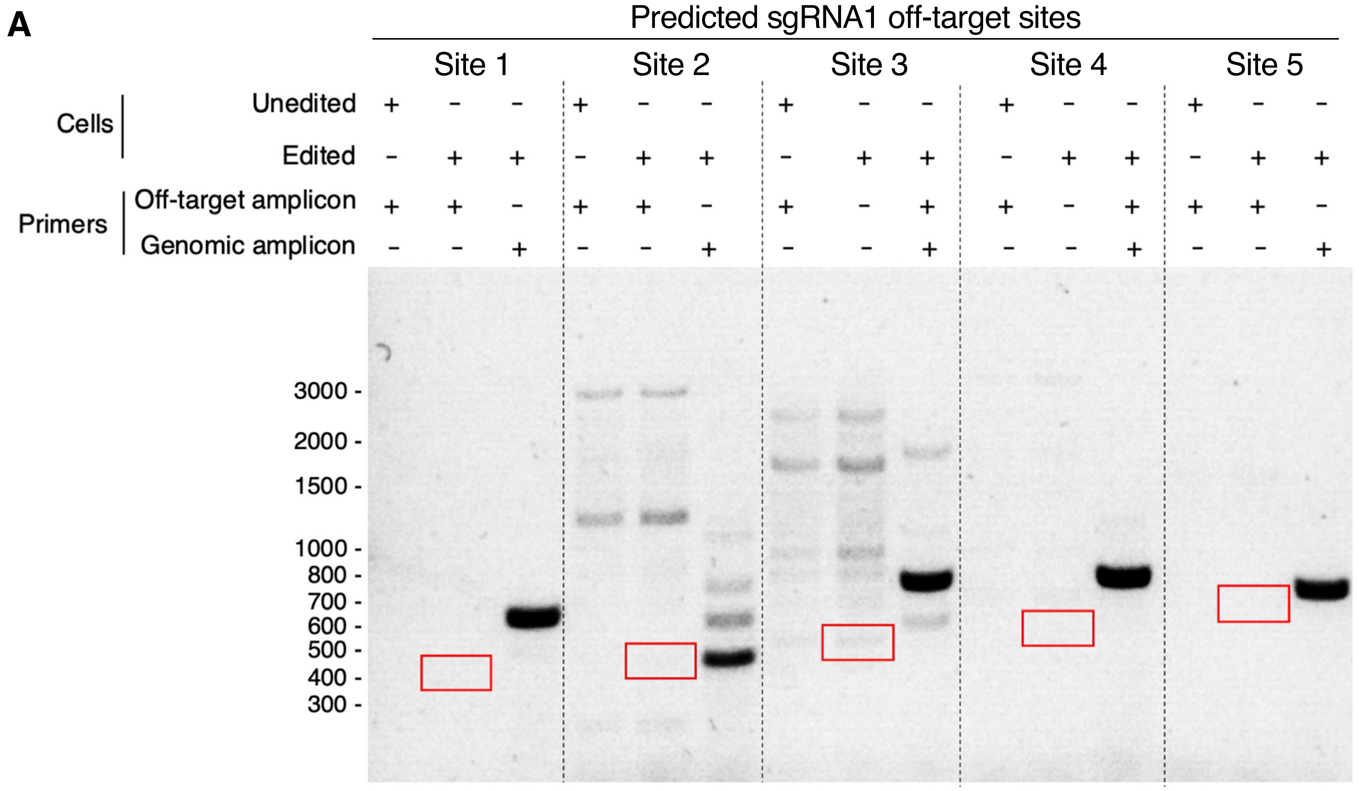
A
Predicted sgRNA1 off-target sites
Site 1
Site 2
Site 3
Site 4
Site 5
Cells
Unedited
Edited
Primers
Off-target amplicon
Genomic amplicon
3000 -
2000 -
1500 -
1000 -
800 -
700 -
600 -
500 -
400 -
300 -

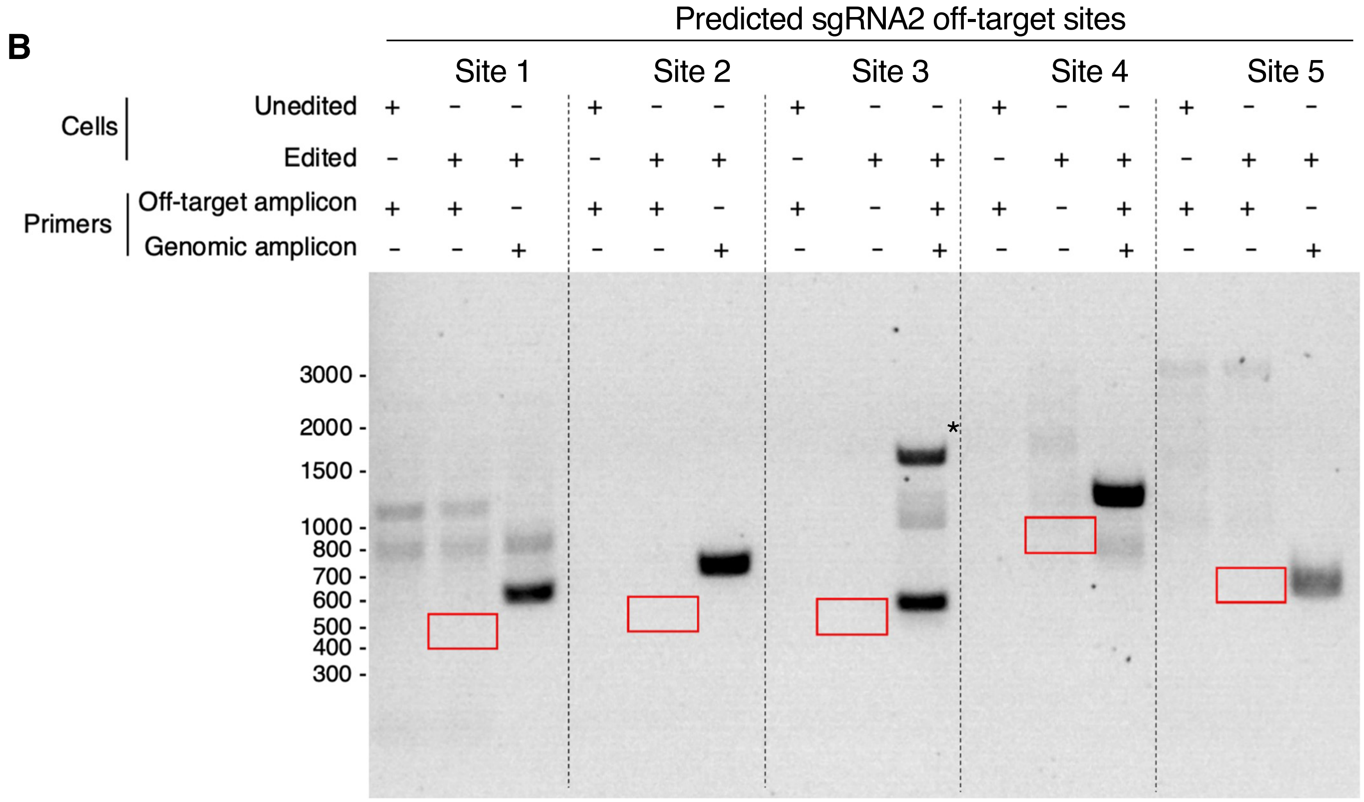
B
Predicted sgRNA2 off-target sites
Site 1
Site 2
Site 3
Site 4
Site 5
Cells
Unedited
Edited
Primers
Off-target amplicon
Genomic amplicon
3000 -
2000 -
1500 -
1000 -
800 -
700 -
600 -
500 -
400 -
300 -
*

 © The Author(s)

◀ **Figure EV2. PCR detection of potential off-target editing in AAVS1 loci edited cells.**

Top 5 predicted off-target sites for sgRNA1 and sgRNA2 were examined. Red box indicate the size of the expected PCR product if off-target integration occurred. * indicate non-specific band amplicon.

© The Author(s)

A

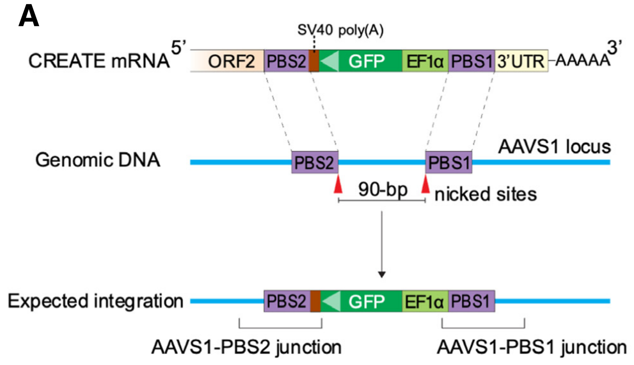

C

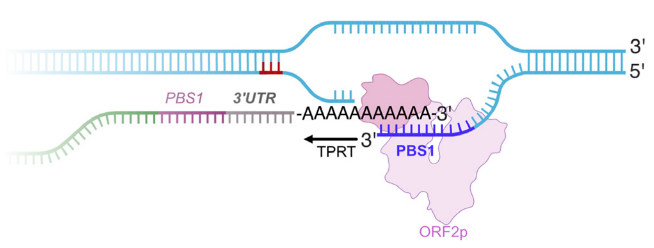

B

Top 5 indel type at AAVS1-PBS1 junction (17 bp PBS, Exp 1)

indel type	observation
EF1α PBS1 3'UTR AAVS1 PBS1; 67-bp addition	Addition of 3'UTR, 35-bp AAVS1 genomic sequence, and PBS1 duplication
EF1α PBS1 3'UTR polyA PBS1; 54-bp addition	Addition of 3'UTR, 11-bp polyA, and PBS1 duplication
EF1α PBS1 3'UTR; 34-bp addition	Addition of 3'UTR and 15-bp unmapped sequence
EF1α PBS1 3'UTR polyA; 61-bp addition	Addition of 3'UTR, 11-bp polyA, and 28-bp unmapped sequence
EF1α PBS1 3'UTR PBS1; 29-bp addition	Addition of 3'UTR and PBS1 (truncated) duplication

Top 5 indel type at AAVS1-PBS1 junction (17 bp PBS, Exp 2)

indel type	observation
EF1α; 74-bp deletion	Deletion of entire PBS1 and 50-bp of EF1a region
EF1α PBS1; 38-bp deletion	8-bp deletion of PBS1 and 30-bp in EF1a region
EF1α PBS1 3'UTR AAVS1 PBS1; 46-bp addition	Addition of 3'UTR, 15-bp AAVS1 genomic sequence, and PBS1 duplication
EF1α PBS1 3'UTR; 40-bp addition	Addition of 3'UTR and 22-bp unmapped sequence
EF1α PBS1 3'UTR polyA; 48-bp addition 68-bp deletion	Addition of 3'UTR and 25-bp polyA, and 68-bp deletion in AAVS1 locus

Figure EV3. Analysis of the most frequently observed indels at PBS1 and PBS2 junctions.

(**A**) A diagram showing expected transgene integration at AAVS1 locus. (**B**) Diagrams and descriptions of the most frequently observed indel patterns at PBS1 junctions in two independent experiments. (**C**) Potential mechanisms of alternative TPRT initiation that explain the observed indels.

 © The Author(s)

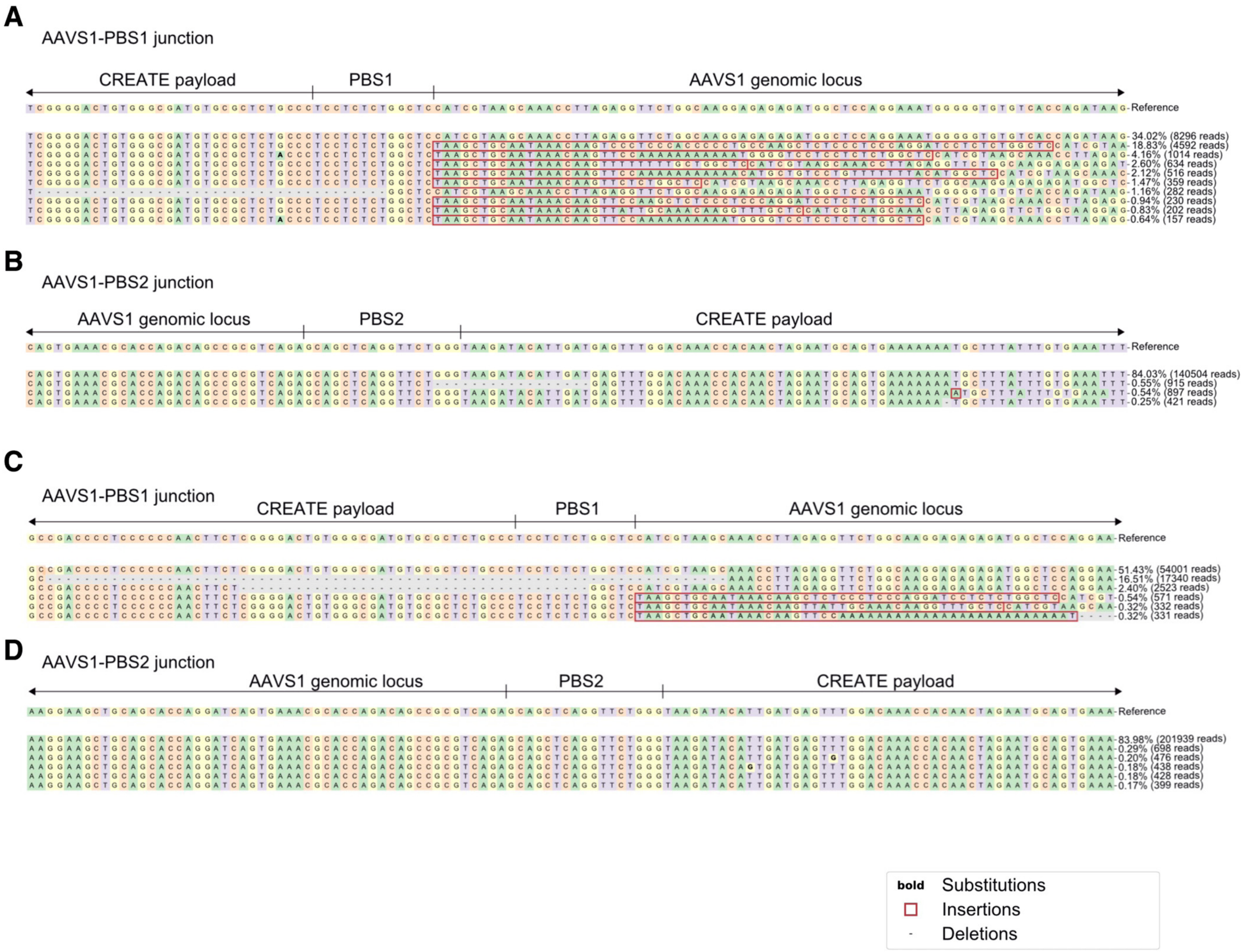

Figure EV4. Allele plots of PBS1 and PBS2 junctions from NGS analysis of AAVS1 locus edited cells.

(**A**) and (**B**) are from AAVS1 (17 bp PBS) Exp1 edited cells. (**C**) and (**D**) are from AAVS1 (17 bp PBS) Exp2 edited cells.

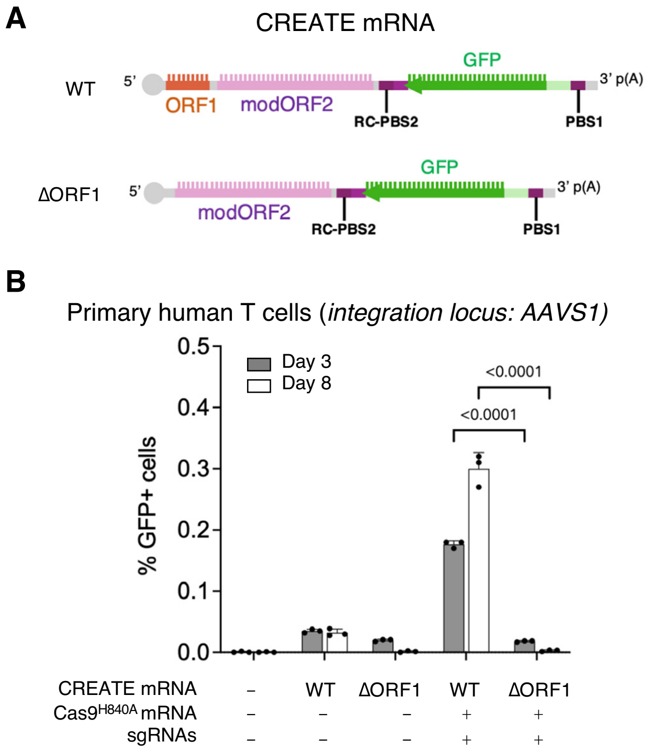

Figure EV5. ORF1 is necessary for CREATE editing in T cells.

(**A**) Diagrams of CREATE mRNA used in the experiment. (**B**) Deletion of ORF1 from CREATE mRNA (ΔORF1) abolished CREATE-mediated integration of GFP payload in primary T cells. Statistical analysis was performed using two-way ANOVA with Dunnett's multiple comparisons test comparing each sample against the sample with WT CREATE mRNA, nCas9^{H840A} mRNA and sgRNAs. Data are mean ± SD ($n = 3$ biological replicates). Experiments were repeated twice. Exact p values: <0.0001 (WT vs ΔORF1, Day 3), <0.0001 (WT vs ΔORF1, Day 8).

© The Author(s)
